# Formyl Peptide Receptor 2 Activation Ameliorates Dermal Fibrosis and Inflammation in Bleomycin-Induced Scleroderma

**DOI:** 10.3389/fimmu.2019.02095

**Published:** 2019-09-03

**Authors:** Gyu Tae Park, Yang Woo Kwon, Tae Wook Lee, Seong Gyu Kwon, Hyun-Chang Ko, Moon Bum Kim, Jae Ho Kim

**Affiliations:** ^1^Department of Physiology, Pusan National University School of Medicine, Yangsan-si, South Korea; ^2^Department of Dermatology, Pusan National University School of Medicine, Yangsan-si, South Korea; ^3^Research Institute of Convergence Biomedical Science and Technology, Pusan National University Yangsan Hospital, Yangsan-si, South Korea

**Keywords:** systemic sclerosis, scleroderma, fibrosis, Fpr2, WKYMVm

## Abstract

Systemic sclerosis is a profibrotic autoimmune disease mediated by the dysregulation of extracellular matrix synthesis. Formyl peptide receptor 2 (Fpr2) is a G protein-coupled receptor that modulates inflammation and host defense by regulating the activation of inflammatory cells, such as macrophages. However, the role of Fpr2 in the development and therapy of scleroderma is still unclear. The present study was conducted to investigate the effects of Fpr2 activation in the treatment of scleroderma fibrosis. We found that intradermal administration of WKYMVm, an Fpr2-specific agonist, alleviated bleomycin-induced scleroderma fibrosis in mice and decreased dermal thickness in scleroderma skin. WKYMVm-treated scleroderma skin tissues displayed reduced numbers of myofibroblasts expressing α-smooth muscle actin, Vimentin, and phosphorylated SMAD3. WKYMVm treatment attenuated macrophage infiltration in scleroderma skin and reduced the number of M2 macrophages. The therapeutic effects of WKYMVm in scleroderma-associated fibrosis and inflammation were completely abrogated in Fpr2 knockout mice. Moreover, WKYMVm treatment reduced the serum levels of inflammatory cytokines, such as tumor necrosis factor-α, and interferon-γ, in the scleroderma model of wild-type mice but not in Fpr2 knockout mice. These results suggest that WKYMVm-induced activation of Fpr2 leads to alleviation of fibrosis by stimulating immune resolution in systemic sclerosis.

## Introduction

Systemic sclerosis or scleroderma is a multiple autoimmune and inflammatory connective tissue disease, resulting in extensive tissue fibrosis in multiple organs. Prominent skin and organ fibrosis is a hallmark feature of scleroderma and is accompanied by fibro-proliferative vasculopathy and immune dysfunction (1, 2). Activated fibroblasts or myofibroblasts, characterized by increased expression of α-smooth muscle actin (α-SMA) and Vimentin, are the key effector cells in scleroderma. Differentiation of fibroblasts into myofibroblasts increases the production of extracellular matrix components, such as collagen, with subsequent development of fibrosis (1, 3). Fibroblast recruitment and differentiation to myofibroblasts are regulated by a combination of autocrine and paracrine profibrotic mediators. Despite significant therapeutic advancements in the treatment of scleroderma, no effective therapy is currently available.

It has been reported that differentiation of tissue-resident fibroblasts to myofibroblasts is promoted by pro-inflammatory mediators which are secreted by macrophages and monocytes (4). Activated macrophages are classified into two major subtypes, M1, and M2 phenotypes (5). M1 type macrophages release pro-inflammatory cytokines, such as TNF-α, interleukin-1β, interleukin-12, and interleukin-23 during inflammation (6). Whereas, M2 macrophages, which are pro-fibrotic/anti-inflammatory, suppress inflammation, and play a key role in tissue fibrosis by secreting TGF-β (7). In fibrosis, inflammatory cytokines induce activation of M2 macrophages expressing transforming growth factor-β (TGF-β), which activate fibroblast differentiation to myofibroblasts (7–9). Accumulating evidence suggests that scleroderma is initiated by chronic inflammation, and that increased infiltration and sustained activation of immune cells are responsible for the extensive fibrosis in scleroderma (3) Moreover, it has been reported that scleroderma can be alleviated by resolution of inflammatory responses (8).

The formyl peptide receptors (FPRs), belonging to the G protein-coupled receptor family, play an important functional role in host defense and inflammation by regulating the activation of phagocytes (10). FPRs are mainly expressed in phagocytes, such as neutrophils, monocytes, and macrophages, and are also functionally expressed in different cell types, such as fibroblasts (11). Three FPRs (FPR1, FPR2/ALX, and FPR3) have been identified in humans, and Fpr1 and Fpr2 have been found in mice as counterparts to human FPR1 and FPR2, respectively, (12). An increasing body of evidence suggests that FPR2 plays a key role in immune resolution. It has been reported that FPR2 is activated by various ligands, such as pro-resolving eicosanoids (lipoxins and resolvins), and a synthetic hexapeptide, Trp-Lys-Tyr-Met-Val-D-Met-NH2 (WKYMVm). It was recently reported that three FPR receptors are overexpressed in scleroderma fibroblasts compared with that in normal fibroblasts (13, 14). Moreover, WKYMVm treatment has been shown to promote differentiation of fibroblasts to myofibroblasts and matrix deposition *in vitro*, suggesting a deteriorating effect of Fpr2 activation in scleroderma. However, the role of Fpr2 in the progression of scleroderma remains elusive.

In the present study, we explored the effect of the Fpr2 agonist WKYMVm on fibrosis and inflammation in the scleroderma animal model, and clarified the role of Fpr2 in WKYMVm-induced therapeutic effect on scleroderma using Fpr2 knockout mice.

## Materials and Methods

### Materials

BLM was purchased from Tokyo Chemical Industry chemicals (Tokyo, Japan). The synthetic peptide, WKYMVm, was synthesized at Anygen (Kwangju, Republic of Korea), and its purity was estimated to be >98%. Anti-α-SMA antibody (ab5694) and anti-CD163 antibody (ab182422) were purchased from Abcam PLC (Cambridge, UK). Anti-CD68 antibody (MCA1957GA) was purchased from AbD Serotec (Raleigh, NC, USA). Anti-NOS2 antibody (SC-650) was purchased from Santa Cruz Biotechnology, Inc. (Dallas, TX, USA). Anti-phospho-SMAD3 antibody (44-246G), Anti-Arginase-I antibody (PA5-29645), Anti-TLR2 antibody (PA5-20020) was purchased from Thermo Fisher Scientific (Waltham, MA, USA). Anti-Vimentin Antibody (MAB-2105SP), Recombinant TGF-β1 (240-B-002) protein was purchased from R&D Systems (Minneapolis, MN, USA). Mouse enzyme-linked immunosorbent assay (ELISA) kits for TNF-α (430904), and INF-γ (430804) were purchased from BioLegend (San Diego, CA, USA). QuickZyme Total Collagen Assay kit was purchased from QuickZyme Biosciences (Leiden, Netherlands).

### Scleroderma Animal Model

BLM powder was dissolved in PBS buffer at a concentration of 1 mg/mL and sterilized by filtration. To establish the scleroderma animal model, C57BL6/J wild-type and Fpr2 knockout mice (6-weeks-old, weighing 22–24 g, male) were subcutaneously injected with 100 μL BLM solution (1 mg/mL) at a single location on the shaved backs (1 cm^2^) of mice for 6 weeks using a 27-gauge needle. The injection was carried out daily for 3 weeks to initially induce scleroderma, followed by subsequent administration of 100 μL PBS containing both BLM (1 mg/mL) and WKYMVm (1 μM) for an additional 3 weeks. As a control experiment, BLM solution without WKYMVm was injected into the scleroderma skin tissues. The therapeutic effects of WKYMVm on scleroderma were examined by histological analysis of the scleroderma skin tissues of mice.

### Histological Analysis and Measurement of Total Collagen Content

For histological analysis of scleroderma tissues, animals were sacrificed, and tissue samples were excised. The skin samples were fixed in 4% paraformaldehyde overnight, and embedded in paraffin. The specimens were sectioned and stained with hematoxylin & eosin (H&E) for determination of dermal thickness, and five areas in one section were randomly selected for this purpose. For determination of the amount of collagen deposition, tissue sections were stained with Masson's Trichrome staining. The images of stained tissue sections were scanned using Axio Scan.Z1 (Carl Zeiss Microscopy, Germany) at ×100 magnification. Using Image J, dermal thickness distance between the epidermal-dermal junction and the dermal-subcutaneous fat junction was measured on H&E-stained images. Collagen density in skin specimens was quantified by using Image J analysis of Masson's Trichrome staining. Collagen deposition in scleroderma skin tissues was measured by a hydroxyproline assay kit (QuickZyme Total Collagen Assay kit) according to the manufacturer's protocol.

### Immunohistochemistry

Formalin fixed, paraffin-embedded skin sections were stained with antibodies against α-SMA, Vimentin, and phospho-SMAD3 for staining of myofibroblasts. Anti-ILB4 antibody and anti-CD68 antibody were used for staining of endothelial cells and macrophages, respectively. Anti-NOS2 and anti-TLR2 antibodies were used for staining of M1 macrophages, and M2 macrophages were stained by anti-CD163, and anti-Arginase-I. The tissue specimens were then incubated with Alexa Fluor 488 goat anti-rabbit, Alexa Fluor 568 goat anti-rabbit, or Alexa Fluor 488 goat anti-rat antibody, followed by washing and mounting in Vectashield medium containing 4′,6-diamidino-2-phenylindole (DAPI) for staining of nuclei. The stained sections were visualized under a laser confocal microscope (Olympus FluoView FV1000). The numbers of α-SMA^+^ILB4^−^, Vimentin^+^, and Vimentin^+^p-SMAD3^+^ myofibroblasts, and the numbers of CD68^+^ macrophages, M1 type (CD68^+^NOS2^+^ and CD68^+^TLR2^+^), M2 type (CD68^+^Arginase-1^+^ and CD68^+^CD163^+^) macrophages were quantified in high-power field using ImageJ software. Four randomly selected microscopic fields from three serial sections in each tissue block were examined by two independent observers blinded to the experimental conditions.

### ELISA Analysis

To measure the levels of inflammatory cytokines in the peripheral blood of mice, blood was collected from scleroderma and therapeutic mice by cardiac puncture using a 27G syringe and allowed to clot at 4°C overnight. Serum was collected after removal of the clot and centrifuged at 1,000 × g for 30 min in 4°C. The levels of TNF-α and INF-γ were assayed using an ELISA kit (BioLegend) according to the manufacturer's instructions. The absorbance values were measured at 450 nm by an ELISA reader and interpolated with a standard curve.

### Statistical Analysis

The results of multiple observations are presented as mean ± SD. Student's two-tailed unpaired *t*-test was used to determine statistical significance of two groups. For multivariate data analysis, group differences were assessed with one-way or two-way ANOVA, followed by Scheffé's *post hoc* test.

## Results

### WKYMVm Treatment Reduces Fibrosis in Bleomycin-Induced Scleroderma

**Figure 1:**
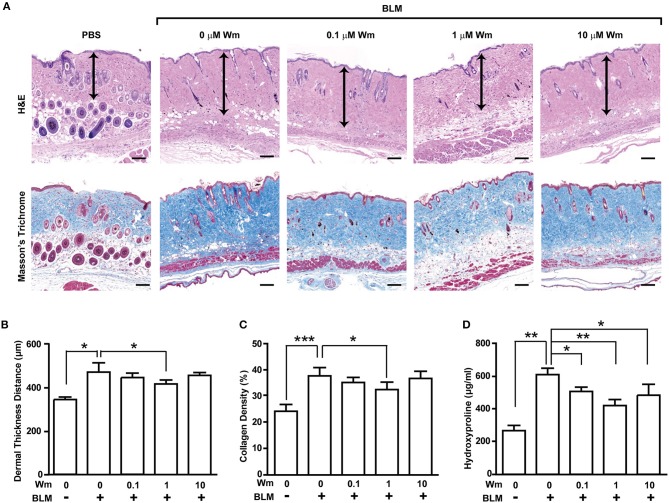
Dose-dependent effects of WKYMVm treatment on tissue fibrosis in a scleroderma mice model. **(A)** Dose-dependent effect of WKYMVm (Wm) on BLM-induced scleroderma. The BLM-induced scleroderma mice were daily treated with the increasing dose of Wm for additional 3 weeks, and skin sections were stained using H&E and Masson's Trichrome staining kits. Dermal layer between the epidermal-dermal junction and the dermal-fat junction was indicated with an arrow on H&E-stained sections. Dermal thickness **(B)** and collagen density **(C)** were quantified from the H&E and Masson's Trichrome data, respectively. **(D)** Effect of Wm on the BLM-induced increase of hydroxyproline content in skin. The levels of hydroxyproline in skin specimens were determined as described in Materials and Methods section. The data are shown as the mean ± SD (*n* = 8 per groups). *p < 0.05, **p < 0.01, ***p < 0.001.

**Figure 2:**
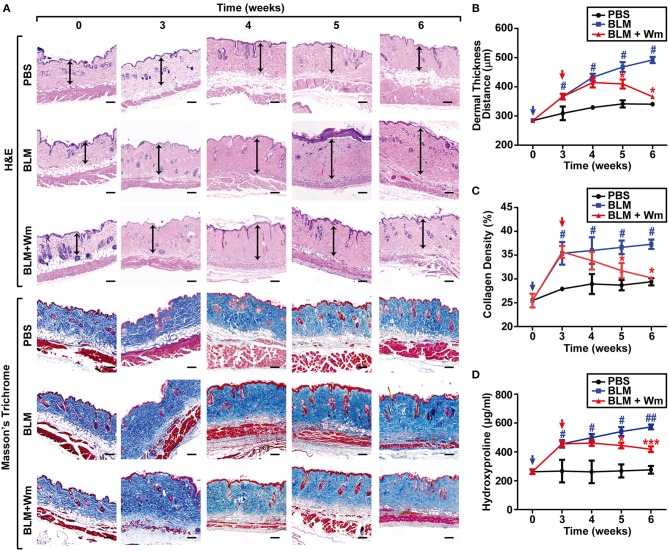
Time-dependent effects of WKYMVm treatment on tissue fibrosis in scleroderma mice model. **(A)** Time dependence of WKYMVm-induced repair of scleroderma. The BLM-induced scleroderma mice were treated without (BLM) or with 1 μM Wm (BLM + Wm) for the indicated time periods. The mock-treated (PBS) and the scleroderma (BLM or BLM + Wm) skin specimens were stained using H&E and Masson's Trichrome staining kits. Dermal layer between the epidermal-dermal junction and the dermal-fat junction was indicated with an arrow on H&E-stained sections. Dermal thickness **(B)** and collagen density **(C)** were quantified from the H&E and Masson's Trichrome staining data, respectively. **(D)** The effects of Wm on the levels of hydroxyproline content in skin specimens were determined. Data are shown as the mean ± SD (*n* = 8). ^#^*p* < 0.05, ^*##*^*p* < 0.01 vs. PBS; *p < 0.05, ***p < 0.001 vs. BLM.

To explore the effect of Fpr2 activation in scleroderma, we established a scleroderma murine model by subcutaneous injection of bleomycin (BLM) for 3 weeks. Subcutaneous injection of BLM alone resulted in increased dermal thickness and collagen deposition that paralleled with a reduction of subcutaneous adipose layer, which was replaced by connective tissues containing collagen. To explore the effects of Fpr2 activation on therapy of scleroderma, the BLM-induced scleroderma mice were subcutaneously co-administrated with both BLM, and WKYMVm for additional 3 weeks. Subcutaneous injection of WKYMVm significantly reduced the dermal thickness associated with BLM-induced scleroderma at 1 μM concentration (Figures 1A,B). Moreover, BLM-induced scleroderma skin exhibited excessive deposition of thick collagen fibers extending into the subdermal adipose tissue and subcutaneous injection of BLM and WKYMVm markedly alleviated skin fibrosis and collagen deposition, as demonstrated by Masson's Trichrome staining (Figures 1A,C). Consistently, the levels of hydroxyproline, which is associated with collagen as an indicator of the severity of fibrosis (15), increased in BLM-induced scleroderma skin, and administration with WKYMVm significantly reduced the BLM-increased hydroxyproline content in skin (Figure 1D). WKYMVm treatment significantly decreased the BLM-induced dermal thickness, collagen density, and hydroxyproline levels at 0.5 μM and maximally reduced those values at 1 μM concentration (Figure S1). Moreover, the BLM-induced increase of dermal- thickness and collagen deposition was time-dependently alleviated by administration of WKYMVm for additional 3 weeks (Figures 2A–D).

**Figure 3:**
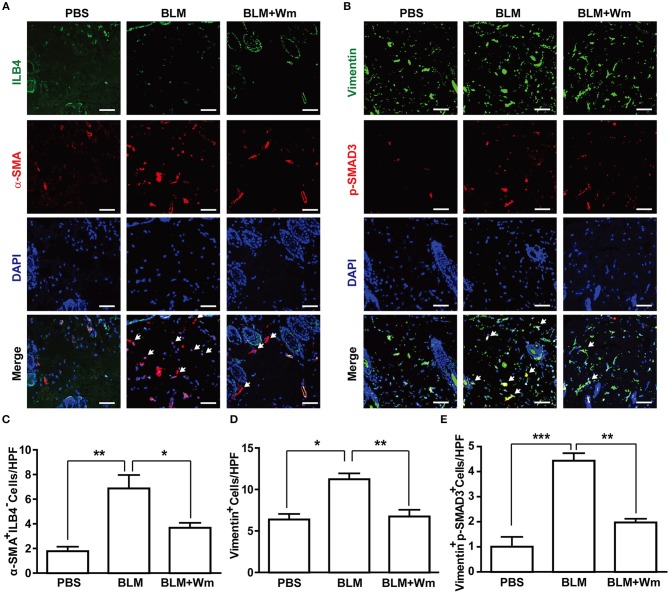
Effects of WKYMVm treatment on myofibroblast differentiation in scleroderma. **(A)** WKYMVm-induced inhibition of myofibroblast differentiation in scleroderma. The BLM-induced scleroderma mice were treated with or without 1 μM Wm for 3 weeks, and the mock-treated (PBS) and the scleroderma (BLM or BLM + Wm) skin specimens were immunostained with antibodies against α-SMA and ILB-4. **(B)** The skin specimens were stained with antibodies against Vimentin and phospho-SMAD3. Scale bar = 50 μm. The numbers of α-SMA^+^ILB-4^−^ **(C)**, Vimentin^+^ **(D)**, and Vimentin^+^phospho-SMAD3^+^ **(E)** cells were quantified under a high-power field. Data represent mean ± SD (*n* = 8 per group). *p < 0.05, **p < 0.01, ***p < 0.001.

It has been reported that fibroblast activation or myofibroblast differentiation plays a key role in the progression of scleroderma (1). To explore the effects of WKYMVm on myofibroblast differentiation, we performed immunostaining of the scleroderma skin specimens with antibodies against α-SMA and Vimentin, which are markers for myofibroblasts (16, 17). α-SMA is expressed in not only myofibroblasts but also blood vessels expressing ILB4, an endothelial marker. Therefore, the number of α-SMA^+^ILB4^−^ cells was counted for determination of myofibroblast differentiation. BLM treatment increased the number of α-SMA^+^ILB4^−^ cells, and WKYMVm treatment abrogated the BLM-induced increase of α-SMA^+^ILB4^−^ cells (Figures 3A,C). Moreover, the number of Vimentin-positive myofibroblasts also increased in BLM-treated skin, and WKYMVm treatment decreased the BLM-induced increase of Vimentin-positive cells (Figures 3B,D). TGF-β-dependent SMAD3 phosphorylation has been reported to play a key role in myofibroblast differentiation during the progression of fibrotic diseases (18). Therefore, we next quantified the number of Vimentin- and p-SMAD3-double positive myofibroblasts. The number of Vimentin^+^p-SMAD3^+^ myofibroblasts increased in the BLM-induced scleroderma model, and WKYMVm treatment markedly reduced the BLM-induced increase of Vimentin^+^p-SMAD3^+^ cell number (Figures 3B,E). Consistently, BLM treatment increased the mRNA level of tissue inhibitor of metalloproteinase-1, which plays a key role in scleroderma-associated fibrosis (19), and WKYMVm treatment reduced the BLM-induced expression of tissue inhibitor of metalloproteinase-1 (Figure S2A).

To explore whether Fpr2 activation directly regulates the differentiation of fibroblasts to α-SMA-positive myofibroblasts, we next examined the effects of WKYMVm on myofibroblast differentiation induced by TGF-β1, which is a well-known ligand that induces myofibroblast differentiation (20). TGF-β1-induced α-SMA expression in fibroblasts was not affected by treatment with WKYMVm (Figure S3), suggesting that WKYMVm does not directly inhibit TGF-β1-induced differentiation of fibroblasts to myofibroblasts. Taken together, these results suggest that WKYMVm-induced activation of Fpr2 alleviated BLM-induced dermal thickness, collagen deposition, and myofibroblast differentiation in scleroderma skin.

### WKYMVm Treatment Reduces Inflammation in BLM-Induced Scleroderma

**Figure 4:**
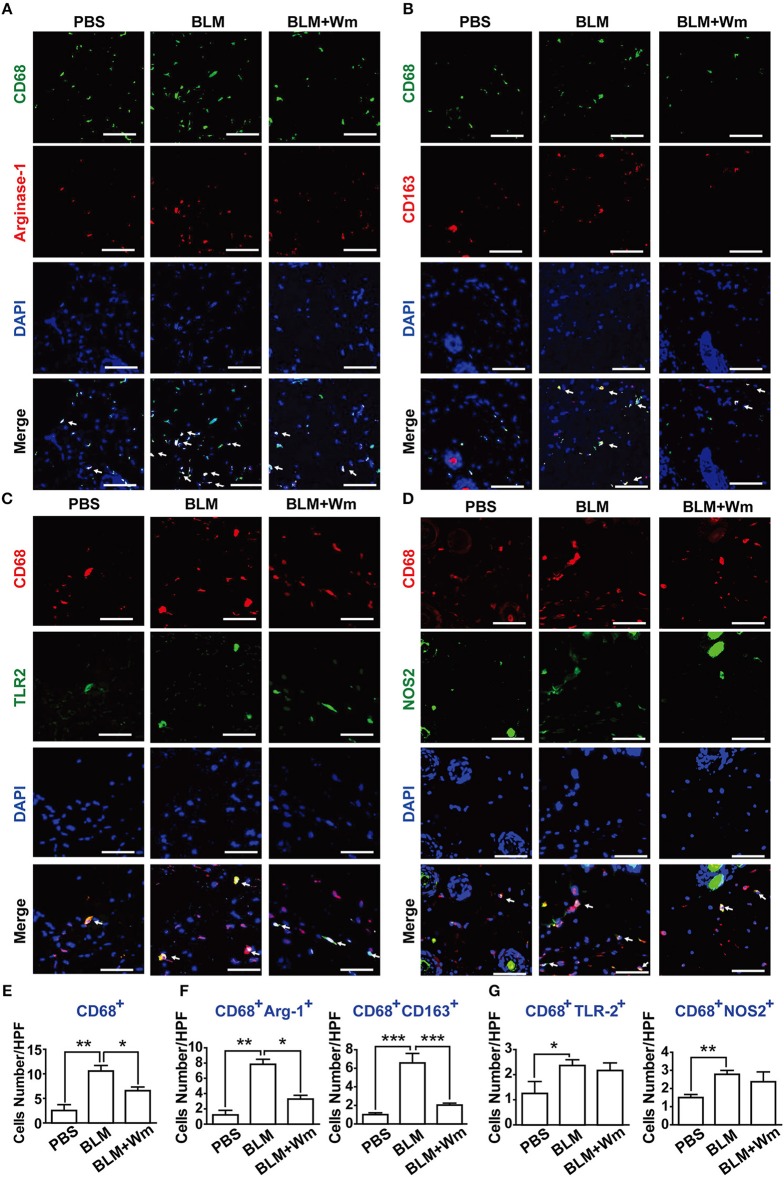
Effects of WKYMVm treatment on macrophage polarization and inflammation in scleroderma. The BLM-induced scleroderma mice were treated with or without Wm for 3 weeks. The mock-treated (PBS) and scleroderma (BLM or BLM+Wm) skin specimens were stained with anti-CD68 antibody together with anti-Arginase-1 **(A)**, anti-CD163 **(B)**, anti-TLR2 **(C)**, or NOS2 **(D)** antibodies. Nuclei was stained with DAPI, and overlaid images are shown. Scale bar = 50 μm. The numbers of CD68^+^ macrophages **(E)**, M2 type macrophages (**F**: CD68^+^Arginse-1^+^ and CD68^+^CD163^+^ cells), M1 type macrophages (**G**: CD68^+^TLR2^+^ and CD68^+^NOS2^+^ cells) were counted under high-power field. Data represent mean ± SD (*n* = 8 per group). *p < 0.05, **p < 0.01, ***p < 0.001.

Infiltration of inflammatory cells, including macrophages, and chronic inflammation have been reported to induce fibrotic disease (3). To explore the effect of WKYMVm on scleroderma-associated infiltration and activation of inflammatory cells, we assessed the levels of macrophage infiltration into the skin in the BLM-induced scleroderma model. The number of CD68-positive macrophages increased in the BLM-induced scleroderma skin compared with that in PBS-treated control specimens; however, this effect was suppressed by WKYMVm treatment (Figures 4A–E). In fibrotic disease, remodeling/profibrotic (M2) macrophages, which express CD163 or Arginase-I, have been implicated in pulmonary fibrosis disease (3, 9). The numbers of CD68^+^ macrophages, CD68^+^NOS2^+^ inflammatory M1 macrophages, and CD68^+^Arginase-1^+^ M2 macrophages in skin were increased within 1 week after subcutaneous administration of BLM with earlier increase of M1 type than M2 type macrophages (Figure S4). Therefore, we quantified the numbers of M2 macrophages, which include CD68^+^CD163^+^ or CD68^+^Arginase-I^+^ populations, and M1 macrophages including CD68^+^NOS2^+^ and CD68^+^TLR2^+^ populations in scleroderma skin. The numbers of CD68^+^CD163^+^ or CD68^+^Arginase-I^+^ cells were greatly increased in BLM-induced skin, and the BLM-induced increase of M2 macrophage populations was considerably attenuated by WKYMVm treatment for additional 3 weeks (Figures 4A,B,F). Moreover, the numbers of M1 macrophages including CD68^+^NOS2^+^ and CD68^+^TLR2^+^ populations increased in BLM-induced scleroderma skin; however, the BLM-induced increase of M1 macrophage (CD68^+^NOS2^+^ and CD68^+^TLR2^+^) populations was not significantly affected by WKYMVm treatment (Figures 4C,D,G). WKYMVm treatment time-dependently decrease the number of M2 macrophages but not M1 macrophages (Figure S5).

### The Role of Fpr2 in WKYMVm-Induced Alleviation of Scleroderma Fibrosis

**Figure 5:**
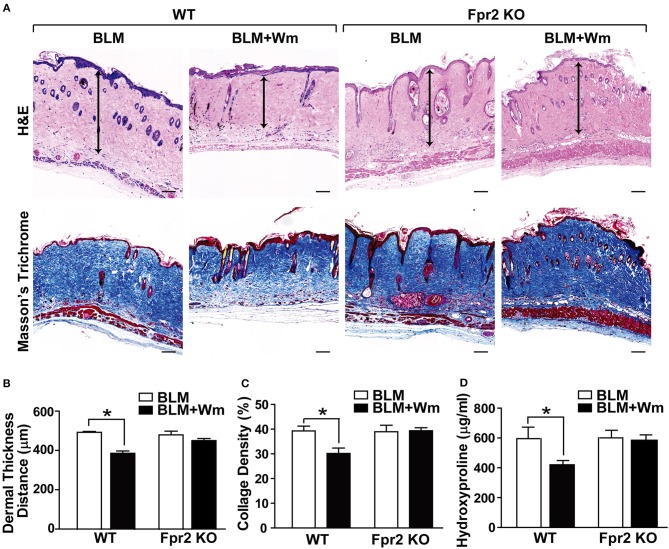
The role of Fpr2 in WKYMVm-induced alleviation of dermal fibrosis in scleroderma. **(A)** Wild type and Fpr2 KO mice were daily injected with BLM for 3 weeks, followed by co-administration of BLM with or without 1 μM Wm for additional 3 weeks. Skin specimens of BLM-induced scleroderma mice were stained with H&E and Masson's trichrome stain. Scale bar = 100 μm. Dermal thickness **(B)** and collagen density **(C)** were quantified from the H&E and Masson's Trichrome data, respectively. **(D)** The levels of hydroxyproline content in skin specimens were determined by using a hydroxyproline assay kit. Data are shown as the mean ± SD (*n* = 8). *p < 0.05.

To clarify the role of Fpr2 in WKYMVm-induced alleviation of scleroderma, we examined the therapeutic effects of WKYMVm on BLM-induced scleroderma in Fpr2 knockout mice. In contrast to the WKYMVm-induced decrease of fibrotic area in the scleroderma model of wild-type mice, WKYMVM treatment did not alleviate the BLM-induced dermal thickness in Fpr2 knockout mice (Figures 5A,B). Moreover, the BLM-induced deposition of thick collagen fibers in the dermal tissues was significantly reduced by WKYMVm treatment in wild type mice but not in Fpr2 knockout mice (Figures 5A,C). Consistently, the increased level of hydroxyproline in the BLM-induced scleroderma skin was not affected by WKYMVm treatment in Fpr2 KO mice, in contrast to significant decrease of hydroxyproline content in the BLM-treated skin of wild type mice (Figure 5D).

**Figure 6:**
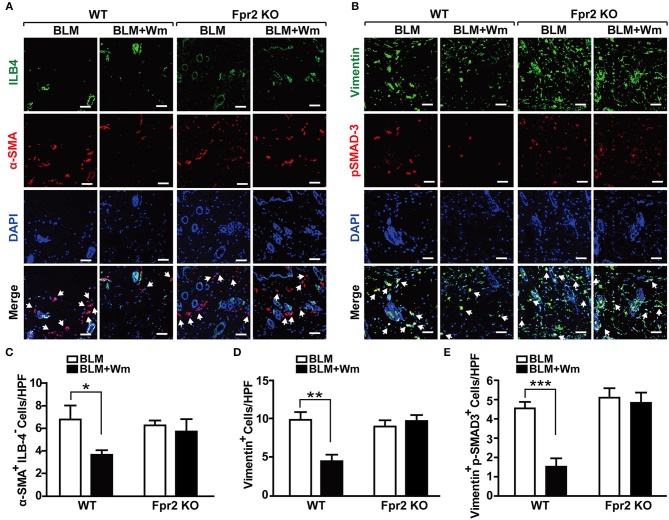
The role of Fpr2 activation in the myofibroblast differentiation associated with scleroderma. **(A)** The BLM-induced scleroderma model of wild type and Fpr2 KO mice were daily injected with or without Wm for 3 weeks, followed by staining of the skin specimens with antibodies against α-SMA and ILB-4. **(B)** The skin specimens were stained with antibodies against Vimentin and phospho-SMAD3. Scale bar = 50 μm. The numbers of α-SMA^+^ILB-4^−^ **(C)**, Vimentin^+^ **(D)**, and Vimentin^+^phospho-SMAD3^+^ **(E)** cells were quantified under a high-power field. Data are shown as mean ± SD (*n* = 8 per group). *p < 0.05, **p < 0.01, ***p < 0.001.

In order to confirm these results, we next explored the role of Fpr2 in WKYMVm-induced regulation of myofibroblast differentiation using Fpr2 knockout mice. In contrast to the WKYMVm-induced decrease of α-SMA^+^ILB4^−^ myofibroblasts in BLM-induced scleroderma skin of wild type mice, WKYMVm treatment had no significant effect on the number of α-SMA^+^ILB4^−^ myofibroblasts in the BLM-induced scleroderma skin of Fpr2 KO mice (Figures 6A,C). Furthermore, WKYMVm treatment did not reduce the numbers of Vimentin^+^ or Vimentin^+^p-SMAD3^+^ myofibroblasts in the scleroderma skin of Fpr2 KO mice, in contrast to the significant inhibition of those in the scleroderma skin of wild type mice (Figures 6B,D,E). These results suggest that Fpr2 mediates WKYMVm-induced reduction of myofibroblast differentiation in scleroderma.

### The Role of Fpr2 in WKYMVm-Induced Immune Resolution in Scleroderma Mice

**Figure 7:**
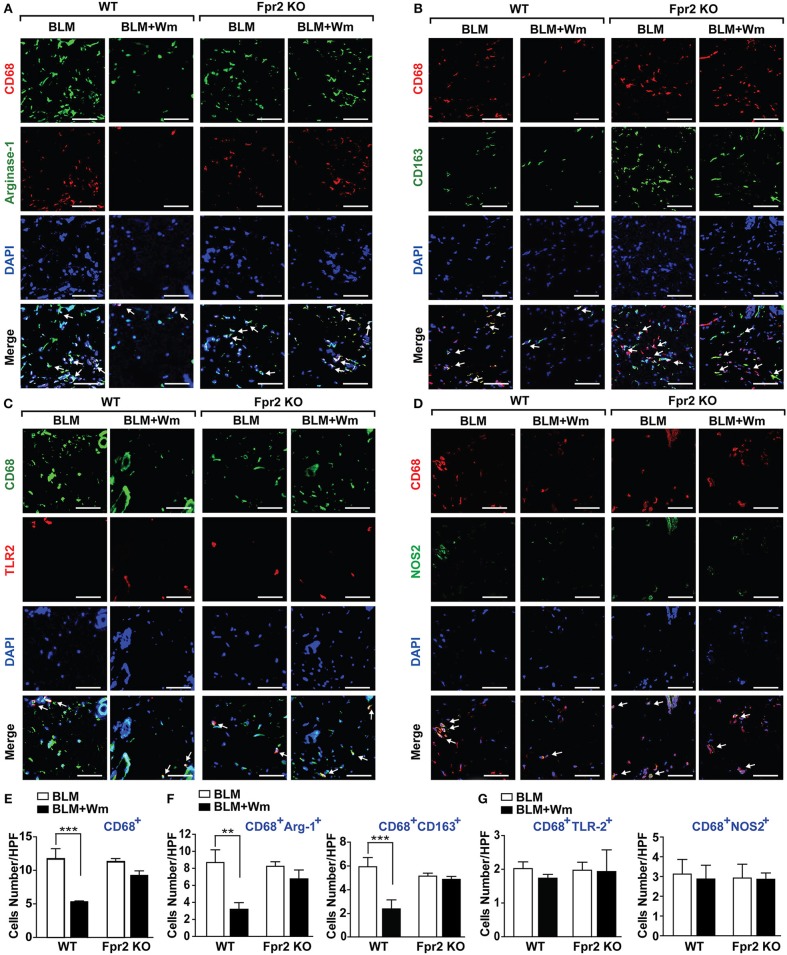
The role of Fpr2 in WKYMVm-induced decrease of M2 macrophages in scleroderma skin. The BLM-induced scleroderma skin of wild type and Fpr2 KO mice were injected with or without Wm for 3 weeks, followed by staining with anti-CD68 antibody together with anti-Arginase-1 **(A)**, anti-CD163 **(B)**, anti-TLR2 **(C)**, or NOS2 **(D)** antibodies. Nuclei was stained with DAPI and overlaid images are shown. Scale bar = 50 μm. The numbers of CD68^+^ macrophages **(E)**, M2 type macrophages (**F**: CD68^+^Arginse-1^+^ and CD68^+^CD163^+^ cells), M1 type macrophages (**G**: CD68^+^TLR2^+^ and CD68^+^NOS2^+^ cells) were counted under high-power field. Data represent mean ± SD (*n* = 8 per group). **p < 0.01, ***p < 0.001.

To explore the role of Fpr2 in WKYMVm-induced alleviation of inflammatory responses in scleroderma mice, we next quantified the number of CD68-positive macrophages in Fpr2 knockout mice. In contrast to the WKYMVm-induced inhibition of CD68-positive macrophage infiltration in the scleroderma skin of wild type mice, WKYMVm treatment had no significant impact on BLM-induced infiltration of macrophages in Fpr2 KO mice (Figures 7A–E). Moreover, WKYMVm treatment did not reduce the numbers of CD68^+^CD163^+^ or CD68^+^Arginase-I^+^ M2 macrophages in scleroderma skin of Fpr2 KO mice, in contrast to the WKYMVm-induced decrease of M2 macrophage levels in that of wild type mice (Figure 7F). Whereas, WKYMVm treatment had no significant impact on the numbers of the CD68^+^NOS2^+^ and CD68^+^TLR2^+^ M1 macrophages in not only wild type mice but also Fpr2 knockout mice (Figure 7G). These results suggest that WKYMVm-induced activation of Fpr2 led to inhibition of macrophage activation and decrease of M2 macrophages in scleroderma skin.

**Figure 8:**
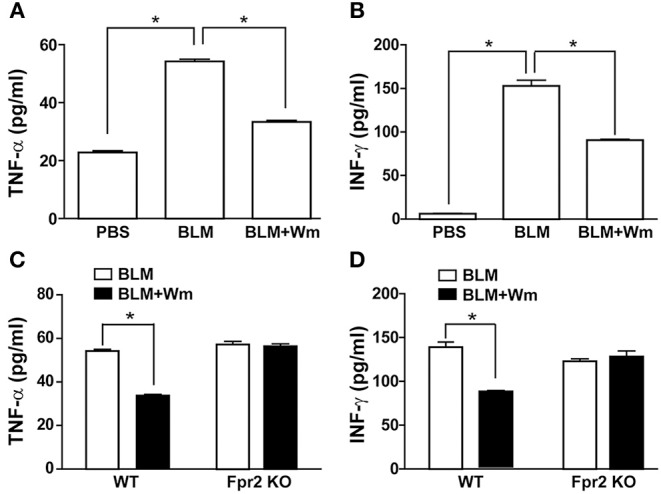
The role of Fpr2 in WKYMVm-induced immune resolution in scleroderma. **(A,B)** Wm-induced inhibition of systemic inflammation in scleroderma. The BLM-induced scleroderma mice model were treated with or without Wm for 3 weeks, and the serum levels of TNF-α **(A)** and INF-γ **(B)** in the mock-treated (PBS) and the scleroderma mice (BLM or BLM+Wm) were determined by ELISA assay. **(C,D)** The role of Fpr2 in Wm-induced suppression of systemic inflammation in scleroderma. The BLM-induced scleroderma model of wild type and Fpr2 KO mice were daily injected with or without Wm for 3 weeks, and the serum levels of TNF-α **(C)**, and INF-γ **(D)** in the scleroderma mice were quantified by ELISA analysis. Data represent mean ± SD (*n* = 8 per group). *p < 0.05.

To explore the immune resolution effect of FPR activation, we measured the levels of inflammatory cytokines in the serum of BLM-induced scleroderma mice. BLM-induced scleroderma mice exhibited increased serum levels of TNF-α and INF-γ, and WKYMVm treatment alleviated the BLM-induced increase of TNF-α and INF-γ (Figures 8A,B). Consistently, BLM treatment increased the mRNA level of INF-γ in scleroderma tissues, and WKYMVm treatment reduced BLM-induced increase of INF-γ mRNA levels (Figure S2B). The WKYMVm-induced reduction of TNF-α and INF-γ levels was observed in the serum of BLM-induced scleroderma model of wild type mice, but not in Fpr2 knockout mice (Figures 8C,D). These results indicate that Fpr2 plays a key role in WKYMVm-induced immune resolution in scleroderma.

## Discussion

In this study, we showed that WKYMVm-induced activation of Fpr2 reduced the skin fibrosis which is associated with BLM-induced scleroderma. Intradermal injection of WKYMVm decreased dermal thickness and collagen deposition in the scleroderma skin of wild type mice, whereas this therapeutic effect was completely abrogated in Fpr2 knockout mice. Fpr2 in mouse has been reported to be activated by not only WKYMVm, but also various structurally diverse ligands, including annexin A1, LL37, lipoxins, and resolvins (21). In accordance with the present study, several reports have suggested that Fpr2 activation leads to alleviation of tissue fibrosis associated with chronic inflammation. Lipoxin was shown to exhibit pro-resolution effect on inflammation and inhibit growth factor-induced fibroblast proliferation and collagen synthesis (22). Moreover, 17®-resolvin D1 ameliorated BLM-induced pulmonary fibrosis by attenuating alveolar infiltration of neutrophils and inflammatory responses (23). These results support the findings of the present study, suggesting the therapeutic role of Fpr2 activation in the treatment of scleroderma.

The pro-resolving effects of Fpr2 ligands include limiting leukocyte trafficking and activation *in vitro* and *in vivo* (24) as well as stimulating efferocytosis (25), granulocyte apoptosis (26), and leukocyte egress (27). One of the downstream Fpr2 signaling events involved in the pro-resolving effects is the suppression of calcium-sensing kinase calcium-calmodulin-dependent protein kinase and subsequent inhibition of p38 mitogen-activated protein kinase (MAPK) phosphorylation in murine bone marrow-derived cells (28, 29). Another possible mechanism associated with the Fpr2-mediated immune resolution is receptor desensitization, which plays a key role in the regulation of G protein-coupled receptor signaling and trafficking (30). Recent studies have demonstrated that FPR2 agonists, lipoxin A4 and some of ureidopropanamide derivatives, induced FPR2 internalization and desensitization (25, 31). Moreover, it has been reported that compound 43, a non-peptidyl agonist for mouse Fpr1 and Fpr2, inhibited neutrophil migration in mice by inducing cross-desensitization of CXCR2, a chemoattractant receptor (32). In addition, some FPR2 agonists including WKYMVm have been reported to induce cross-desensitization of CCR5 and CXCR4 (33, 34). Therefore, it is likely that inhibition of inflammatory signaling and desensitization of chemoattractant receptors may be play a key role in the WKYMVm-induced resolution of chronic inflammation.

Systemic scleroderma is a chronic autoimmune disease that causes systemic inflammation. In this study, we showed that BLM-induced scleroderma stimulated infiltration of CD68-positive macrophages into scleroderma skin, and the serum levels of the inflammatory cytokines, TNF-α and INF-γ, were up-regulated in the scleroderma model; however, WKYMVm treatment attenuated these effects. Accumulating evidence suggests that activation of FPR2 led to alleviation of chronic inflammatory diseases (35). In dextran sodium sulfate-treated ulcerative colitis animal model, WKYMVm administration alleviated mucosa destruction and shortened colon by decreasing production of interleukin-23, a pro-inflammatory cytokine, and TGF-β1 (36). WKYMVm treatment prevented the development of Th1 and Th17 cell responses and inhibited the production of inflammatory cytokines (37). Moreover, WKYMVm treatment attenuated hyperoxia-induced lung injury and lung inflammation in newborn mice (38). WKYMVm treatment decreased the numbers of inflammatory cells including CD68-positive macrophages and the levels of inflammatory cytokines in the lungs of hyperoxic mice. These results suggest that WKYMVm-induced activation of Fpr2 attenuates chronic inflammation and concomitant production of inflammatory cytokines.

It has been shown that WKYMVm treatment increases the expression of Fpr2 in scleroderma fibroblasts and upregulation of Fpr2 in scleroderma fibroblasts fosters the switch to myofibroblasts (13, 14). However, lipoxin A4 has been shown to inhibit TGF-β1-dependent profibrotic activity in human lung myofibroblasts (39). Furthermore, lipoxin A4 inhibited the proliferation of human lung fibroblasts induced by connective tissue growth factor (40). In our study, WKYMVm treatment led to decreased production of myofibroblasts positive for α-SMA, Vimentin, and phospho-SMAD3 through an Fpr2-dependent mechanism in the scleroderma model. However, WKYMVm treatment did not directly affect TGF-β1-induced differentiation of fibroblasts to α-SMA-positive myofibroblasts, suggesting that WKYMVm-induced activation of Fpr2 indirectly alleviates myofibroblast differentiation in the BLM-induced scleroderma model. Macrophage-derived TGF-β stimulates myofibroblasts to produce various extracellular matrix proteins and inhibitors of metalloproteases that are involved in the degradation of matrix proteins (41). Several drugs, such as tamibarotene, glycyrrhizin, and paquinimod have been reported to ameliorate BLM-induced dermal fibrosis and infiltration of macrophages by suppressing M2 polarization of macrophages in BLM-induced scleroderma model (7, 8, 42, 43). In this study, we demonstrated that WKYMVm treatment reduced the number of CD68^+^CD163^+^ or CD68^+^Arginase-I^+^ M2 macrophages in the scleroderma tissues through Fpr2-dependent mechanism. These results suggest that Fpr2 activation alleviates scleroderma by inhibiting M2 macrophage polarization and systemic levels of inflammatory cytokines in scleroderma.

In conclusion, the present study demonstrates that WKYMVm-induced Fpr2 activation relieves fibrosis by inhibiting fibroblast activation, macrophage infiltration and generation of M2 type macrophages, and inflammatory cytokine expression in BLM-induced scleroderma model. These results suggest that Fpr2 ligands including WKYMVm can be useful in the treatment of patients with scleroderma.

## Data Availability

All datasets generated for this study are included in the manuscript/Supplementary Files.

## Ethics Statement

All animal experiments were performed according to protocols approved by the Institutional Animal Care and Use Committee of Pusan National University Institutional Animal Use and Care Committee.

## Author Contributions

GP and JK designed the experiments and wrote the manuscript. GP, YK, TL, and SK performed the experiments. H-CK and MK analyzed data.

### Conflict of Interest Statement

The authors declare that the research was conducted in the absence of any commercial or financial relationships that could be construed as a potential conflict of interest.

## Abbreviations

α-SMA: α-smooth muscle actin
BLM: bleomycin
ELISA: Enzyme-linked immunosorbent assay
FPR2: Formyl peptide receptor 2
IFN-γ: interferon-γ
PBS: phosphate buffered saline
TGF-β1: transforming growth factor-β1
TNF-α: tumor necrosis factor-α
Wm: WKYMVm.
