# Supplementary material for: Formyl Peptide Receptor 2 Activation Ameliorates Dermal Fibrosis and Inflammation in Bleomycin-Induced Scleroderma
Source: Front Immunol. 2019 Sep 3;10:2095. doi: 10.3389/fimmu.2019.02095 (PMC6733889; doi:10.3389/fimmu.2019.02095)
Supplement: Supplementary file 1 [file Data_Sheet_1.PDF]

Supplemental Materials

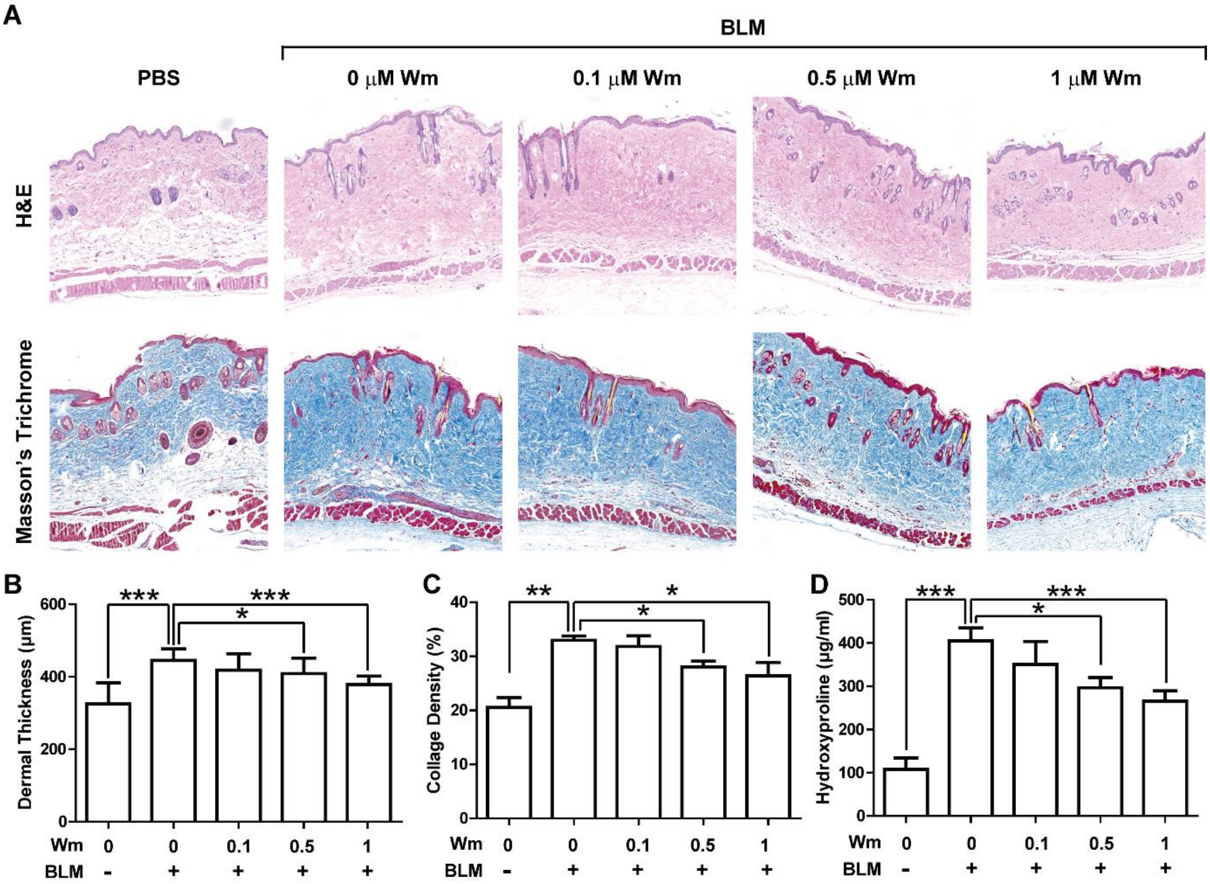

Figure S1. Dose-dependent effects of WKYMVm treatment on tissue fibrosis in a scleroderma mice model. (A) Dose-dependent effect of WKYMVm (Wm) on BLM-induced scleroderma. The BLM-induced scleroderma mice were daily treated with the increasing dose of Wm for additional three weeks, and skin sections were stained using H&E and Masson's Trichrome staining kits. Dermal layer between the epidermal-dermal junction and the dermal-fat junction was indicated with an arrow on H&E-stained sections. Dermal thickness (B) and collagen density (C) were quantified from the H&E and Masson's Trichrome data, respectively. (D) Effect of Wm on the BLM-induced increase of hydroxyproline content in skin. The levels of hydroxyproline in skin specimens were determined as described in Materials and Methods section. The data are shown as the mean ± SD (n=8 per groups). *p<0.05, **p < 0.01, ***p < 0.001.

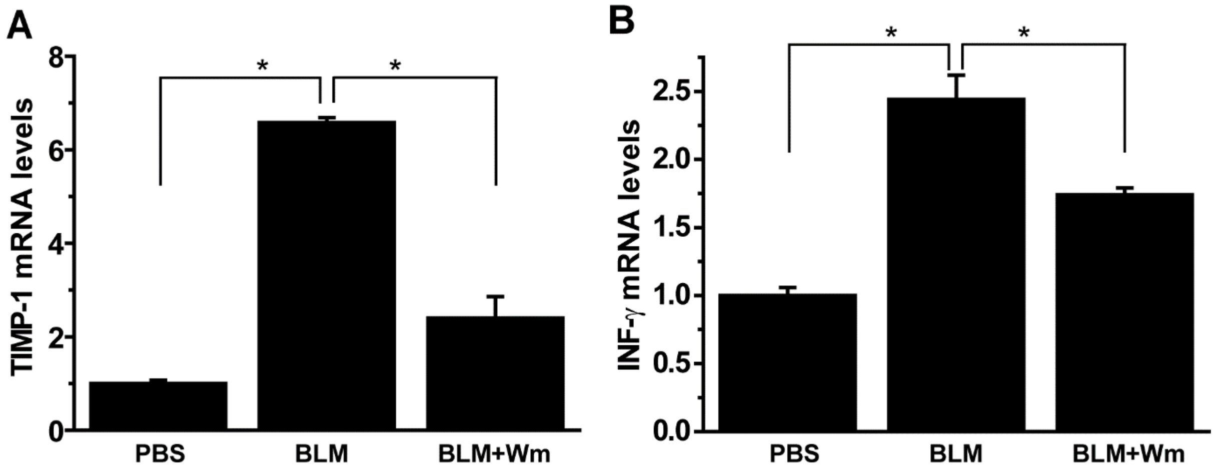

Figure S2. Effects of WKYMVm treatment on mRNA levels of TIMP-1 and INF-γ in a scleroderma mice model. WKYMVm-induced inhibition of TIMP-1 and INF-γ expression in scleroderma skin. The BLM-induced scleroderma mice model were treated with or without Wm for three weeks, and the mRNA levels of TIMP-1 (A) and INF-γ (B) in the skin tissues of the mock-treated (PBS) and the scleroderma mice (BLM or BLM+Wm) were determined by real time RT-PCR analysis. Data are shown as the mean ± SD (n=4).

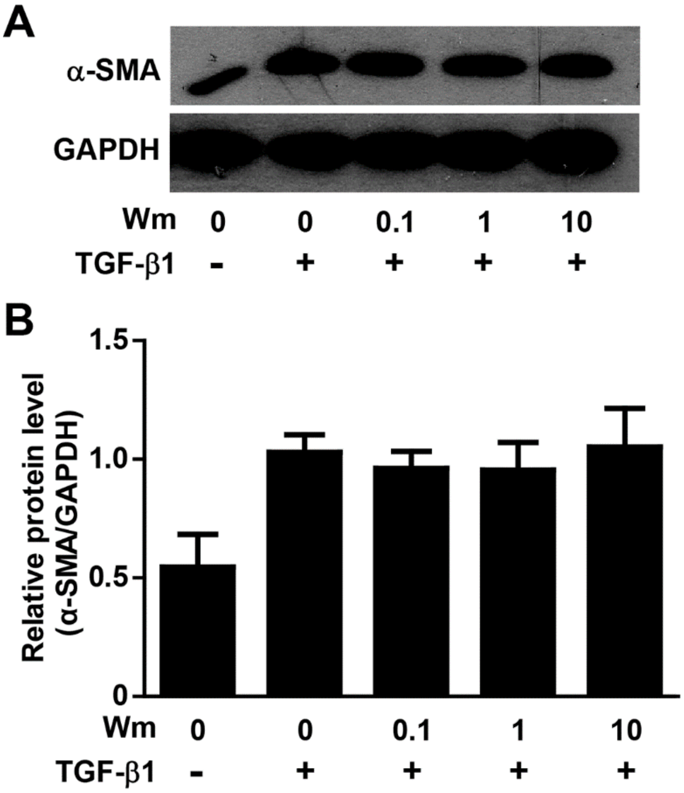

Figure S3. Effects of WKYMVm treatment on myofibroblast differentiation of human dermal fibroblasts. (A) Human dermal fibroblasts were treated with TGF-β1 in the presence of the increasing concentrations of WKYMVm for 2 days. The protein levels of α-SMA and GAPDH were determined by Western blotting. (B) The intensities of protein bands of α-SMA were quantified and normalized to those of GAPDH. The relative levels of α-SMA are shown as the mean ± SD (n=4).

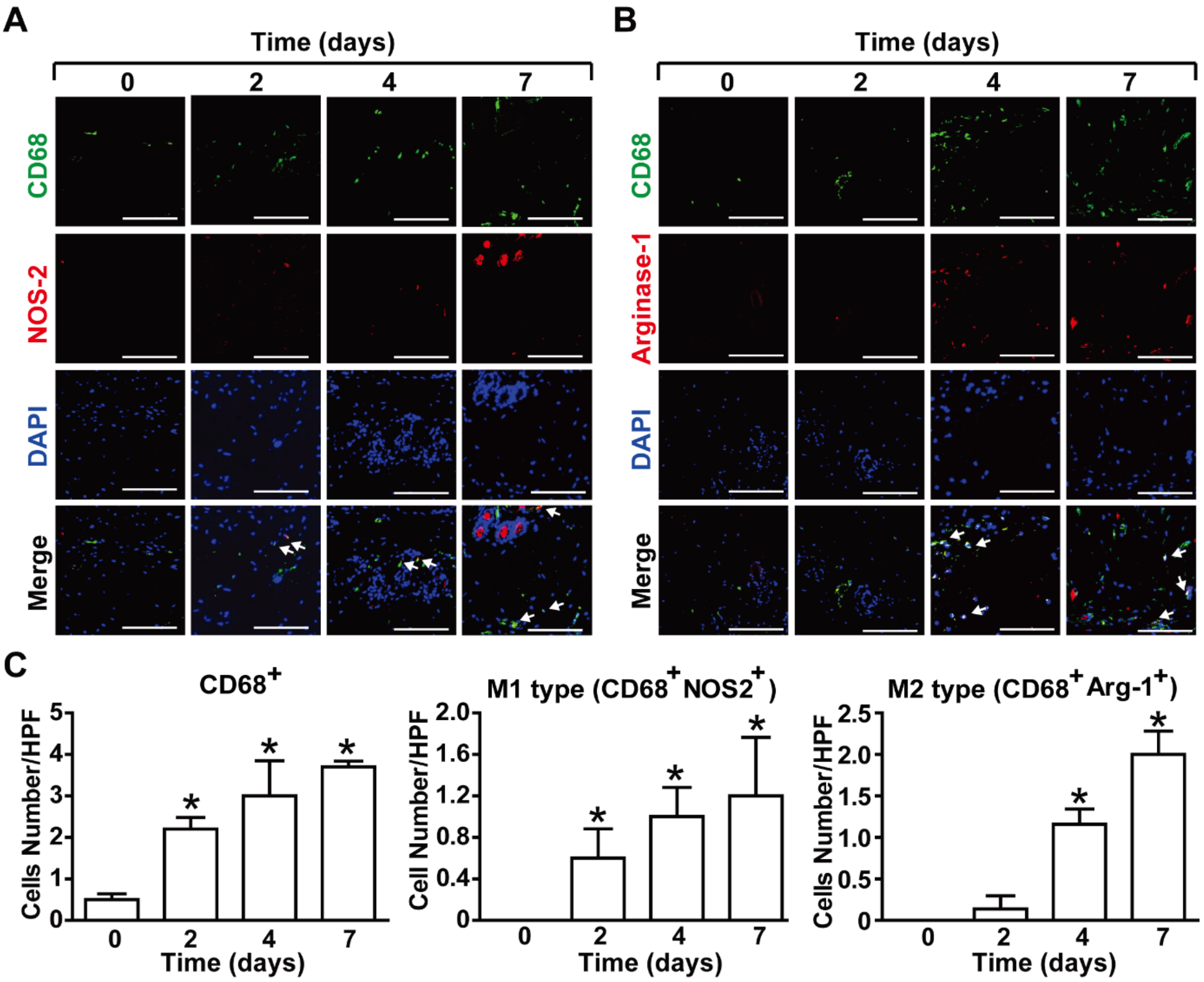

Figure S4. Time-dependent effects of BLM treatment on the numbers of macrophages in skin. (A, B) The mice were treated with BLM (1 mg/mL) for the indicated time periods, and skin specimens were stained with anti-CD68 antibody together with anti-NOS2 (A) or anti-Arginase-1 (B) antibodies. Nuclei was stained with DAPI and overlaid images are shown. Scale bar = 50 μm. (C) The numbers of $CD68^+$ macrophages, M1 type macrophages ($CD68^+NOS2^+$ cells), and M2 type macrophages ($CD68^+$Arginse-1^+ cells) were counted under high-power field. Data represent mean ± SD (n = 8 per group). *, $p < 0.05$ *vs* control (day 0).

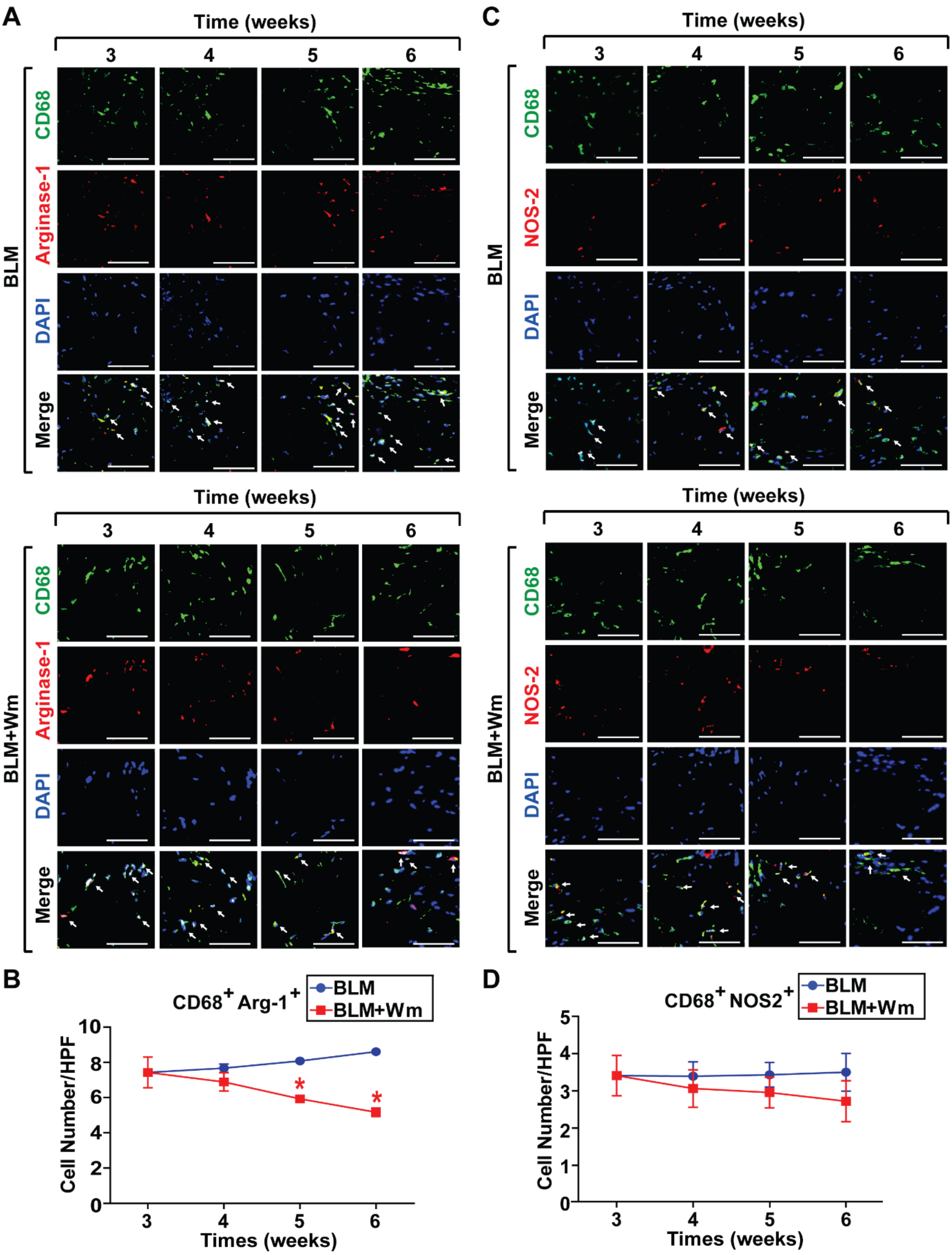

Figure S5. Time-dependent effects of WKYMVm treatment on macrophage polarization and inflammation in scleroderma. The BLM-induced scleroderma mice model were produced by subcutaneous injection of BLM for three weeks, followed by treatment without (BLM) or with 1 µM Wm (BLM+Wm) for additional three weeks. The scleroderma (BLM or BLM+Wm) skin specimens at the indicated time points were stained with anti-CD68

antibody together with anti-Arginase-1 (A) or anti-NOS2 (B) antibodies. Nuclei was stained with DAPI and overlaid images are shown. Scale bar = 50 μm. The numbers of M2 type macrophages ($CD68^{+}Arginse\text{-}1^{+}$ cells) and M1 type macrophages ($CD68^{+}NOS2^{+}$ cells) were counted under high-power field. Data represent mean ± SD (n = 8 per group). *$p < 0.05$ *vs* BLM.

Supplemental Materials and Methods

Cell culture

Human dermal fibroblasts were isolated by mechanical and enzymatic digestion of human juvenile foreskin using a slight modification of a previously described protocol (1). Written informed consent was obtained from all blood donors, and the experimental protocol was approved by the Institutional Review Board of Pusan National University Hospital. After removing the epidermis, the dermal tissue was cut into small pieces and washed three times with phosphate buffered saline (PBS) at room temperature. Thereafter, the pieces were incubated with 0.25% collagenase for 30 min at 37 °C with intermittent shaking. Supernatants were harvested and centrifuged at 300 × *g* for 5 min. The cell pellet was re-suspended in α-minimum essential medium (α-MEM) supplemented with 10% fetal bovine serum (FBS), 50 μg/mL ascorbic acid, 100 U/mL penicillin, and 100 μg/mL streptomycin. Cells were seeded at a density of 1×10^5 cells per well in 6-well culture plates and grown until they reached a subconfluent state in α-MEM containing 10% FBS. Cell quiescence was induced by incubating the cells in serum-free medium for 24 h. The serum-starved cells were treated with appropriate reagents for 4 days, and the expression levels of α-SMA and Glyceraldehyde 3-phosphate dehydrogenase (GAPDH) were determined by western blotting, and mock-treated cells served as negative controls.

Western blotting

Cells were lysed in a lysis buffer (pH 7.4; 20 mM Tris-HCl, 1 mM EGTA, 1 mM EDTA, 10 mM NaCl, 0.1 mM phenylmethyl sulfonyl fluoride, 1 mM Na3VO4, 30 mM sodium pyrophosphate, 25 mM β-glycerol phosphate, and 1% Triton X-100). Lysates were resolved by sodium dodecyl sulfate-polyacrylamide gel electrophoresis (SDS-PAGE) and the proteins

were transferred to nitrocellulose membranes. The proteins were stained with 0.1% Ponceau S solution (Sigma-Aldrich Co. Ltd, St. Louis, MO), blocked with 5% non-fat milk, and immunoblotted with anti-α-SMA and anti-GAPDH antibodies overnight. Bound antibodies were visualized with horseradish peroxidase-conjugated secondary antibodies using an Enhanced Chemiluminescence system (GE Healthcare Life Sciences, Pittsburgh, PA, USA).

Real time polymerase chain reaction analysis

Total cellular RNA was extracted using the TRIzol (T9424, Sigma-Aldrich) method. For RT-PCR analysis, 2 μg of each RNA aliquot were subjected to cDNA synthesis using 200 U of M-MLV reverse transcriptase and 0.5 μg of oligo (dT) 15 primer (C-1101, Promega). cDNA in 2 μl of the reaction mixture was amplified using 0.5 U of GoTaq DNA polymerase (M8298, Promega). Quantitative RT-PCR was performed on an ABI 7500 (Applied Biosystems) sequence detection system with SYBR Green PCR Master Mix (ABS-4309155, Applied Biosystems) according to the manufacturer's instructions. Experiments were performed in triplicate. The data were analyzed using the Δ (Δ CT) method and normalized to GAPDH mRNA levels. The RT-PCR primer sequences are listed as follows. TIMP-1 (forward: 5'-CCAGAGCCGTCACTTTGCTT-3'; reverse: 5'-AGGAAAAGTAGACAGTGTTCAGGCTT-3'), IFN-γ (forward: 5'-TCAAGTGGCATAGATGTGGAAGAA-3'; reverse: 5'-TGGCTCTGCAGGATTTTCATG-3'), GAPDH (forward: 5'-GTGTTCCTACCCCCAATGTGT-3'; reverse: 5'-ATTGTCATACCAGGAAATGAGCTT-3').

References

1. Rittie, L., and Fisher, G.J. Isolation and culture of skin fibroblasts. *Methods Mol Med* (2005). 117, 83-98. doi: 10.1385/1-59259-940-0:083.
